# Patellofemoral outcomes after kinematic versus mechanical alignment in primary total knee arthroplasty: a focused scoping review of head-to-head comparative studies

**DOI:** 10.1302/2633-1462.78.BJO-2026-0079.R1

**Published:** 2026-08-07

**Authors:** Seikai Toyooka, Yutoshi Osaki, Noriaki Arai, Hirotaka Kawano, Takumi Nakagawa

**Affiliations:** 1 Department of Orthopaedic Surgery, Teikyo University School of Medicine, Tokyo, Japan

**Keywords:** Kinematic alignment, Mechanical alignment, Total knee arthroplasty, Patellofemoral, Scoping review, primary total knee arthroplasty, patient-reported outcome measures (PROMs), total knee arthroplasty (TKA), radiological measures, MEDLINE, CINAHL, randomized controlled trials, PFJ, patellar height, patellar resurfacing

## Abstract

**Aims:**

Concerns persist that kinematic alignment (KA) in primary total knee arthroplasty (TKA) may adversely affect the patellofemoral (PF) joint, because most contemporary components were developed under mechanical alignment (MA) principles. This focused scoping review mapped head-to-head comparative clinical evidence on PF outcomes after KA versus MA TKA, and identified key evidence gaps relevant to clinical decision-making.

**Methods:**

A focused scoping review was conducted. PubMed/MEDLINE, the Cochrane Library, and CINAHL were searched from inception to 15 January 2026. We included head-to-head comparative studies directly comparing KA and MA in primary TKA that reported at least one PF-related outcome. PF outcomes were mapped into three domains: 1) PF symptoms and PF-specific patient-reported outcome measures (PROMs); 2) PF-related interventions and complications; and 3) PF imaging/radiological measures. Findings were synthesized descriptively.

**Results:**

A total of 12 studies met the inclusion criteria. Domain A was addressed by only one study and was insufficient for definitive conclusions. Domain B was addressed by six studies; no consistent signal of higher PF-related interventions/complications with KA was identified, and several studies reported numerically fewer PF-related procedures with KA, including lateral retinacular release. Domain C was addressed by ten studies and showed heterogeneous imaging findings; when PF radiological differences were observed, they were not consistently accompanied by worse symptoms or higher PF-related intervention rates. Postoperative rotational alignment was inconsistently assessed.

**Conclusion:**

Current head-to-head comparative evidence is insufficient to determine the definitive PF-specific clinical impact of KA versus MA, mainly because PF-specific PROMs are rarely reported. Although no consistent harm signal was identified, this should not be interpreted as evidence of equivalence. This review primarily defines current evidence gaps, including the need for prespecified PF-specific PROMs, systematic rotational verification, consistent reporting of PF-related interventions/complications, and longer follow-up.

Cite this article: *Bone Jt Open* 2026;7(8):1026–1036.

## Introduction

Kinematic alignment (KA) in primary total knee arthroplasty (TKA) aims to restore the patient’s prearthritic joint lines and limb phenotype, with the intention of reproducing more native knee kinematics than conventional mechanical alignment (MA).^1-3^ Recent work using the Coronal Plane Alignment of the Knee classification has highlighted substantial interindividual variability in native limb alignment and joint-line obliquity.^4^ These observations challenge a ‘one size fits all’ MA target and provide a rationale for KA-based and other personalized alignment strategies aimed at restoring patient-specific alignment and kinematics. As interest in KA and related personalized alignment strategies has increased, so too have concerns regarding the patellofemoral (PF) joint. Most contemporary femoral components were developed and validated under MA principles, and their trochlear geometry and orientation may not fully accommodate the wide interindividual variation in quadriceps line of force and extensor mechanism alignment that KA seeks to restore.^5-7^ This has led to the hypothesis that KA may adversely influence patellar tracking, PF contact mechanics, and, ultimately, PF-related symptoms or complications.^8-10^

PF outcomes after TKA are clinically important because anterior knee pain, PF dissatisfaction, maltracking, and secondary PF procedures can substantially affect patient satisfaction despite otherwise satisfactory tibiofemoral outcomes.^11,12^ However, PF outcome assessment remains inconsistent across the alignment literature. Many comparative TKA studies prioritize global patient-reported outcome measures (PROMs), implant survival, or general radiological alignment, whereas PF-specific symptoms and PF-specific PROMs are infrequently prespecified and often incompletely reported.^2,13,14^ In addition, radiological surrogate measures – such as patellar tilt, patellar shift, patellar height indices, and trochlea-related parameters – are commonly used, although their relationship to clinically meaningful PF symptoms is uncertain.^15-17^ A previous review has examined PF considerations under KA by incorporating heterogeneous study designs, including clinical studies, imaging analyses, simulations, and cadaveric investigations.^18^ While several of these limitations also apply to the current review, our approach differs by restricting eligibility to head-to-head KA-versus-MA comparative studies and mapping PF outcomes by domain, thereby providing a clinician-facing evidence map that complements broader syntheses.^18-20^

The purpose of this focused scoping review was therefore to map the available head-to-head comparative evidence on PF outcomes after KA versus MA in primary TKA. Specifically, we sought to classify reported PF outcomes into clinically relevant domains – PF symptoms and PF-specific PROMs, PF-related interventions and complications, and PF imaging/radiological measures – in order to identify what is currently known, what remains inconsistent, and where the most important evidence gaps persist.

## Methods

### Study design and reporting guideline

This study was conducted as a focused scoping review to map head-to-head comparative evidence on PF outcomes after KA versus MA TKA. A scoping review design was chosen because the aim was to map the breadth, nature, and heterogeneity of the available evidence across multiple PF outcome domains, rather than to generate pooled effect estimates or grade certainty of evidence. We followed the methodological framework proposed by Arksey and O’Malley,^21^ Colquhoun et al,^22^ and the Joanna Briggs Institute for scoping reviews during the conduct and reporting of this study.^23^

### Stage 1: Identifying the research question

Among adults undergoing primary TKA, what head-to-head comparative evidence exists regarding PF outcomes after KA versus MA?

### Stage 2: Identifying relevant studies (information sources and search strategy)

We systematically searched PubMed/MEDLINE, the Cochrane Library, and CINAHL from inception to 15 January 2026. Two authors (ST and YO) independently performed the searches. Because PF symptoms and PF-specific outcomes are often inconsistently indexed and variably described in titles/abstracts, we used a broad alignment-focused search strategy (KA vs MA in TKA) and subsequently identified PF outcomes during full-text review and data charting. A sample PubMed search string was (“total knee arthroplasty” OR “total knee replacement” OR TKA) AND (kinematic alignment OR kinematically aligned OR KA OR restricted kinematic OR adjusted kinematic) AND (mechanical alignment OR mechanically aligned OR MA). We also screened the reference lists of included studies and relevant reviews to identify additional eligible publications. This approach was chosen to maximize sensitivity for KA–MA comparative studies, with PF outcomes subsequently captured during full-text assessment.

### Stage 3: Study selection (eligibility criteria and selection process)

After importing all records into a reference manager, duplicates were removed. Titles and abstracts were screened for eligibility, followed by full-text assessment of potentially relevant articles. The same two authors (ST and YO) independently performed all stages of study selection; disagreements were resolved by discussion and consensus.

Studies were eligible if they met all of the following criteria: 1) population: adults undergoing primary TKA for degenerative knee disease; 2) concept: direct comparison of KA and MA within the same study (head-to-head comparison); 3) outcomes: reported at least one PF-related outcome, including PF symptoms, PF-specific PROMs, PF imaging/radiological measures (e.g. patellar tilt/shift, patellar height indices, trochlear parameters), or PF-related interventions/complications (e.g. lateral retinacular release (LRR), patellar complications, secondary PF procedures); 4) study type: head-to-head comparative studies, including clinical comparative studies (randomized or non-randomized) and imaging-based comparative studies using patient imaging data (e.g. postoperative imaging comparisons or virtual implantation/surgical planning) that directly compare KA and MA; and 5) language: English.

For this review, ‘KA’ was used as an umbrella term encompassing unrestricted KA and KA variants, including restricted/adjusted KA and inverse-restricted KA, as defined by each included study.

We excluded the following: studies without a direct KA versus MA comparison (single-arm KA or MA only); studies comparing implant designs under a single alignment philosophy only; cadaveric or in vitro studies and purely computational simulations not based on patient imaging data and/or without reportable clinical or imaging outcomes; case reports, editorials, letters, and conference abstracts without sufficient outcome data; and non-human studies. No restrictions were applied to implant design, patellar resurfacing strategy, or surgical approach; these variables were captured during data extraction.

### Stage 4: Charting the data (data extraction and outcome domains)

A standardized data-charting form was developed a priori. For each included study, we extracted the setting, study design, sample size, definitions of KA and MA (including boundaries for restricted/adjusted KA, if applicable), surgical technique (manual/navigation/robotic), implant design, patellar resurfacing status, follow-up duration, and PF-related outcomes and their measurement methods (including imaging modality/view, knee flexion angle, and timepoints for radiological outcomes). PF outcomes were classified into three clinically meaningful domains:

**Domain A:** PF symptoms and PF-specific PROMs (e.g. anterior knee pain, Kujala score, PF-related pain/function measures).

**Domain B:** PF-related interventions and complications (e.g. LRR, patellar maltracking requiring intervention, patellar complications, secondary PF procedures, PF-related reoperation).

**Domain C:** PF imaging/radiological measures (e.g. patellar tilt/shift, patellar height indices, trochlear groove parameters, anterior flange coverage/understuffing). We also charted whether each study reported axial-plane parameters (e.g. femoral/tibial component rotation, CT-based rotational measures, or axial surrogates) and whether postoperative axial verification was performed.

Each study could contribute outcomes to more than one domain. When multiple PF outcomes were reported, all relevant outcomes were charted and mapped.

### Critical appraisal

Risk of bias was assessed at the study level to aid interpretation (RoB 2 for randomized trials and ROBINS-I for non-randomized comparative studies),^24,25^ and an overall judgement is reported. Risk-of-bias assessments were not used to exclude studies; the synthesis reflects reported results. Our objective was to map and describe the available head-to-head evidence rather than to generate pooled effect estimates or to exclude studies based on internal validity. Nevertheless, we recorded key design features (e.g. randomization, matching/adjustment, follow-up duration) to support interpretation.

### Stage 5: Collating, summarizing, and reporting the results (synthesis approach)

Given the anticipated heterogeneity in alignment definitions, imaging protocols, and outcome measures, we performed a descriptive synthesis rather than a meta-analysis. We mapped the evidence by domain and summarized, for each outcome, whether KA was reported as similar to, better than, or worse than MA (direction of effect), and whether differences were statistically significant when reported. Clinically meaningful outcomes (Domains A and B) were emphasized, while imaging outcomes (Domain C) were interpreted as surrogate measures that may not directly translate to symptoms.

## Results

### Search results and study overview

**Fig. 1:**
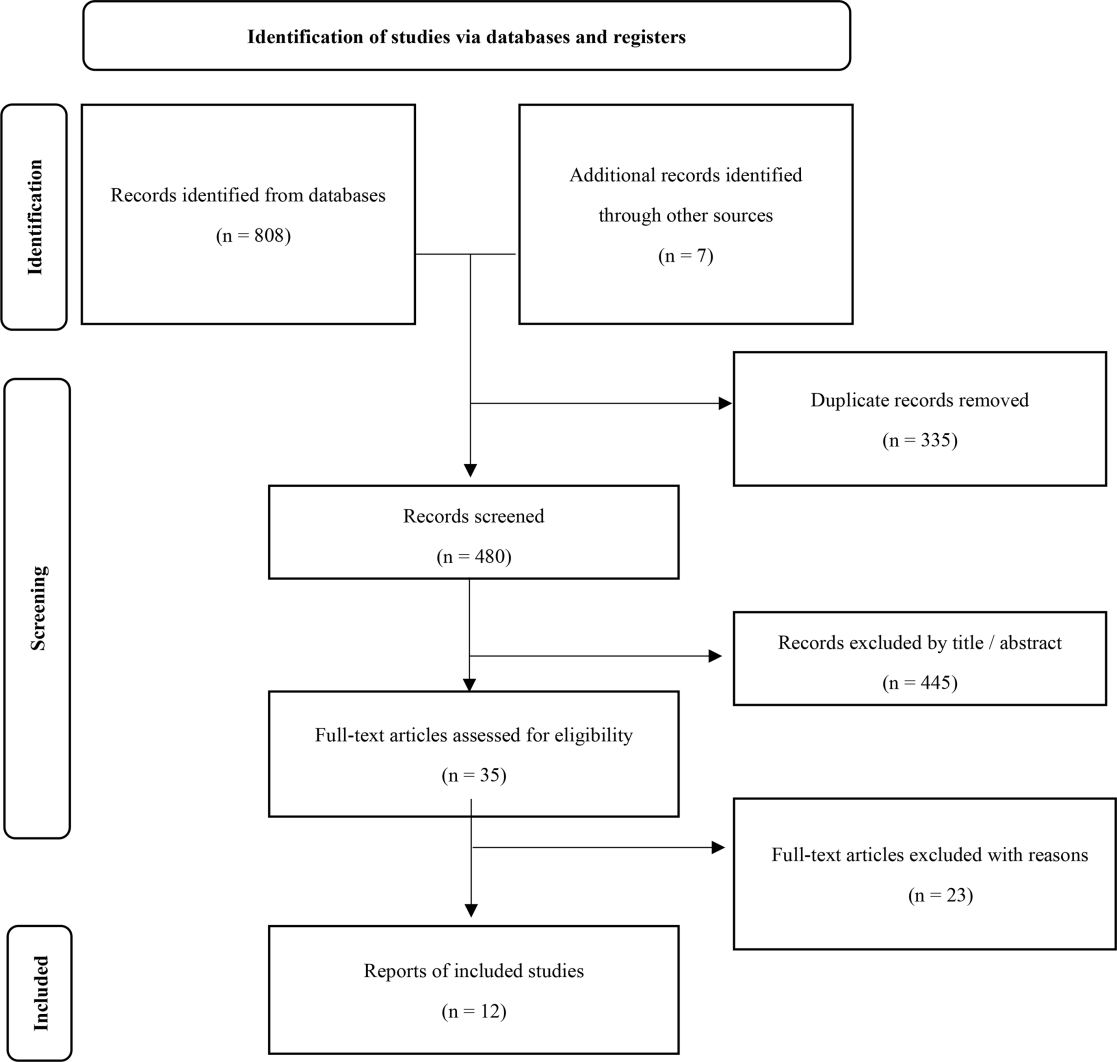
Preferred Reporting Items for Systematic reviews and Meta-Analyses 2020 flow diagram showing the study selection process.

The database search and reference screening identified 12 eligible head-to-head comparative studies reporting at least one PF outcome after KA versus MA TKA (Figure 1). These studies comprised randomized controlled trials (RCTs), registry- or cohort-based comparative studies (including propensity-matched analyses), and imaging-focused comparative reports (Table I).

**Table I.** Characteristics of included head-to-head studies comparing kinematic alignment (KA) and mechanical alignment (MA) total knee arthroplasty (TKA) reporting patellofemoral (PF) outcomes.

Study	Setting	Design	Risk of bias (overall)*	Unit/sample size	KA definition	MA definition	Technique	Implant	Patella resurfacing	Follow-up	PF domains†
Parente et al^26^	Single centre	Retrospective cohort	ROBINS-I: Serious	120 knees (KA 40; adjusted MA 80)	Inverse-restricted KA	Adjusted MA	Manual	Persona PS (Zimmer Biomet); cemented	None (patients without resurfacing)	24 mths	A, C
Sun et al^27^	Single centre	RCT	RoB 2: Some concerns	234 knees (KA 117; MA 117)	Unrestricted KA	MA	Manual	ATTUNE CR (DePuy Synthes)	None (resurfacing excluded)	12 mths	B, C
Wen et al^28^	Single centre	Retrospective cohort	ROBINS-I: Serious	227 knees (KA 102; MA 125)	Unrestricted KA	MA	Manual	Gemini MK II CR (LINK); cemented	NR	6 mths	B, C
McEwen et al^29^	Single centre	RCT	RoB 2: Some concerns	41 patients/ 82 knees (paired)	Restricted KA (one knee)	MA (contralateral knee)	Imageless navigation	Triathlon CR (Stryker); hybrid fixation (cementless femur/cemented tibia)	Selective; 25 (KA) vs 23 (MA) resurfaced	2 yrs	B, C
King et al^30^	Single centre	Comparative cohort	ROBINS-I: Moderate	295 knees (MA 168; KA 127; resurfacing subgroups)	Unrestricted KA	MA	Manual	GMK Sphere (Medacta); cemented, PCL-resecting	Mixed; analyzed resurfaced vs non-resurfaced	1 yr	B, C
Dossett et al^31^	Single centre	RCT	RoB 2: Some concerns	88 knees	Unrestricted KA	MA	PSI (MRI-based)	Vanguard CR (Zimmer Biomet); cemented	All resurfaced	≥ 12 yrs	B
Gibbons et al^32^	Single centre	RCT	RoB 2: Some concerns	99 knees (KA 49; MA 50)	Unrestricted KA	MA	KA: PSI (OtisMed); MA: imageless navigation	Triathlon CR (Stryker); cemented	Selective (intraoperative decision)	10 yrs	B
Koh et al^33^	Single centre	Retrospective cohort (propensity matched)	ROBINS-I: Moderate	186 knees (KA 93; MA 93)	Unrestricted KA	MA	Manual	PFC Sigma CR (DePuy Synthes); cemented	All resurfaced	2 yrs	C
Kim et al^34^	Single centre	Retrospective cohort (propensity matched)	ROBINS-I: Moderate	168 knees (KA 42; MA 126)	Unrestricted KA	MA	Manual	Persona (Zimmer Biomet); cemented	Selective (intraoperative decision)	2 yrs	C
Nagai et al^35^	Single centre	Retrospective cohort	ROBINS-I: Serious	56 knees (KA 28; MA 28)	Unrestricted KA	MA	Navigation (KneeAlign)	NexGen LPS-FLEX (Zimmer Biomet)	All resurfaced	1 yr	C
Elkadi et al^36^	Single centre	Retrospective cohort	ROBINS-I: Serious	256 knees (MA 104; KA 152)	KA	MA	Manual	NR	NR	NR	C
Orsi et al^37^	Single centre	Imaging-based comparative study	N/A (non-clinical imaging study)	61 knees	Unrestricted KA, restricted KA, inverse restricted KA	MA	3D analysis/virtual implantation	Unity CR (Corin)	None (resurfacing excluded)	NR (no clinical follow-up)	C

* Overall risk-of-bias judgement: RoB 2 for randomized trials; ROBINS-I for non-randomized comparative studies; NA for non-clinical imaging-based studies.

† PF domains: A, PF symptoms and PF-specific patient-reported outcome measures; B, PF-related interventions and complications; C, PF imaging/radiological measures.

N/A, not applicable; NR, not reported; PCL, posterior cruciate ligament; PSI, patient-specific instrumentation; RCT, randomized controlled trial.

Across the 12 studies, PF outcomes were mapped into three domains. Domain A (PF symptoms/PF-specific PROMs) was addressed by one study. Domain B (PF-related interventions/complications) was addressed by six studies, including RCTs and retrospective comparisons. Domain C (PF imaging/radiological measures) was addressed by ten studies, with substantial heterogeneity in imaging protocols and measured parameters (e.g. patellar tilt/shift, patellar height indices, PF angles, and trochlea-related measures) (Tables II and III).

**Table II.** Clinical patellofemoral (PF) outcomes (Domains A and B).

Study	Domain A: PF symptoms / PF-specific PROMs	Domain B: PF-related interventions and complications	Key KA vs MA comparison (value)	p-value	Key message (clinical PF relevance)
Parente et al^26^	Kujala score: No between-group difference postoperatively	NR	Mean Kujala score: 86.2 (SD 4.7) (KA) vs 86.6 (SD 6.3) (MA)	0.68	Only included head-to-head PF-specific PROM study; Kujala was comparable between KA and MA
Sun et al^27^	NR	LRR: No signal of higher PF-related complications in KA	LRR: 6.7% (KA) vs 25.4% (MA)	< 0.001	KA was associated with fewer lateral releases, while clinical outcomes were similar or slightly better
King et al^30^	NR	LRR: No signal of higher PF-related complications in KA; revision (including secondary patellar resurfacing) and reoperation rates were similar between groups	LRR: 17% (KA) vs 17% (MA) in non-resurfaced; 12% (KA) vs 13% (MA) in resurfaced	0.924	No evidence that KA increases PF-related interventions/complications versus MA (short-term)
Wen et al^28^	NR	LRR: No signal of higher PF-related complications in KA	LRR: 6.9% (KA) vs 14.4% (MA)	0.071	No statistically significant increase in PF-related intervention with KA
McEwen et al^29^	NR	LRR: No signal of higher PF-related complications in KA; reported complications included an asymptomatic patellar stress fracture (MA)	LRR: 1 (KA) vs 2 (MA)	> 0.999	At ≥ 2 years, clinical outcomes were equivalent; KA did not increase PF-related intervention (and may reduce releases)
Dossett et al^31^	NR	Patella-related minor reoperations and patellar complications: No signal of higher PF-related complications in KA	Patella-related minor reoperations: 5 (KA) vs 3 (MA) Patellar complications: 11% (KA) vs 7% (MA); not statistically different	0.71 (patella-related minor reoperations) NS (patella complications)	Long-term head-to-head RCT data: no clear PF safety signal disadvantage for KA
Gibbons et al^32^	NR	Selective patellar resurfacing: No signal of higher PF-related complications in KA; PF-related reoperations in the KA group included two secondary patellar resurfacings and one traumatic patellar dislocation; overall revision/reoperation-free survivorship was similar at 10 years	Selective patellar resurfacing: 12% (KA) vs 22% (MA)	NS	At 10 years, PF events were uncommon; no clear signal that KA increases PF complications versus MA

KA, kinematic alignment; LRR, lateral retinacular release; MA, mechanical alignment; NR, not reported; NS, not significant; PROMs, patients-reported outcome measures; RCT, randomized controlled trial; TKA, total knee arthroplasty.

**Table III.** Patellofemoral (PF) imaging/radiological outcomes (Domain C) in comparative studies of kinematic alignment (KA) versus mechanical alignment (MA) total knee arthroplasty (TKA).

Study	Imaging modality and timepoints	Axial-plane / rotational assessment	PF imaging outcomes (definitions)	Key KA vs MA comparison (value)	p-value	Key findings (KA vs MA)
Sun et al^27^	Skyline (axial) radiographs 12 months postoperatively	No (not assessed)	Patellar tilt angle (°) Patellar shift (mm)	Postoperative patellar tilt angle: KA 10.1° (SD 1.4°) vs MA 10.6° (SD 1.9°) Postoperative patellar shift: KA 2.2 mm (SD 0.6) vs MA 2.4 mm (SD 0.8)	NS	No significant between-group differences; KA showed slightly smaller tilt/shift
McEwen et al^29^	Skyline (axial) radiographs 2 years postoperatively	No (not assessed)	Patellar tilt angle (°)	Patellar tilt angle: KA 5.7° (SD 5.8°) vs MA 4.4° (SD 4.9°)	NS	No significant difference in patellar tilt
Kim et al^34^	Skyline (axial) radiographs, 3D CT/3D planning for femoral component morphometrics (trochlear groove/anterior flange) 12 months postoperatively	NR (component rotation NR); CT used for component morphometrics	Patellar tilt angle (°) Femoral component morphometric fit: proximal trochlear groove position in theAP and ML directions anterior flange fitting distance (under/overstuffing)	Postoperative patellar tilt angle: KA 2.0° (SD 1.6°) vs MA 0.3° (SD 1.2°) (p < 0.05)	p < 0.05	Morphometrics: KA more often positioned the proximal trochlear groove laterally and had smaller anterior flange mismatch vs MA (study-defined metrics); KA showed greater patellar tilt despite better trochlear/anterior flange fit surrogate measures
Koh et al^33^	Skyline (axial) radiographs, binary classification of tilt 6 months and 2 years postoperatively	No (not assessed)	Patellar tilt positive if > 5° Lateral patellar tilt positive if > 5° lateral	Patellar tilt positive: postoperatively KA 26.9% vs MA 39.8%; 2 years KA 7.5% vs MA 7.5% Lateral patellar tilt positive: postoperatively KA 12.9% vs MA 1.1%; 2 years KA 4.3% vs MA 0%	NS	Lateral tilt was more frequent early after KA, but between-group differences diminished by 2 years
Nagai et al^35^	Skyline (axial) radiographs at 30°, 60°, 90° knee flexion 3 weeks postoperatively	No (not assessed)	Patellar tilt angle (°) and patellar shift (mm/° depending on definition) at different flexion angles	Patellar tilt did not differ between groups; patellar shift was reported to be significantly different at 30° flexion (KA showing greater shift) while other flexion angles were similar	NS	Patellar tilt did not differ between groups; patellar shift was reported to be significantly different at 30° flexion (KA showing greater shift) while other flexion angles were similar
King et al^30^	Skyline (axial) and lateral radiographs 6 weeks and 1 year postoperatively	No (not assessed)	Patellar tilt angle (°) and patella height (CDI)	1 year postoperative patella tilt angle: KA 7.3° (SD 0.5°) vs MA 5.9° (SD 0.4°) 6 weeks postoperative patella height: KA 0.9 (SD 0.2) vs MA 0.9 (SD 0.1)	p = 0.028 (1 year) NS (6 weeks)	KA was associated with higher patellar tilt at 1 year; clinical outcomes were similar in the study
Parente et al^26^	Lateral radiographs 3, 6, 12, 24 months postoperatively	No (not assessed)	Patellar height (Insall index and CDI)	Insall index: KA 1.1 (SD 0.1) vs MA 1.1 (SD 0.2) CDI: KA 1.0 (SD 0.1) vs MA 1.0 (SD 0.2)	NS	No significant differences in Kujala and other outcomes; radiological indices did not indicate PF disadvantage of KA vs MA
Elkadi et al^36^	Lateral radiographs preoperatively and postoperatively	No (not assessed)	Patellar height (ISI, CDI, BPI)	Relative pre–post change (%): ISI MA −4.6% vs KA −1.9% CDI MA −8.7% vs KA +0.6% (p = 0.018) BPI MA −7.4% vs KA −3.6% (NS)	NS (ISI); p = 0.018 (CDI); NS (BPI)	Between-group change favoured KA for CDI (less patella baja signal), though absolute postoperative indices differed between groups
Orsi et al^37^	Patient-derived 3D trochlear morphology with virtual implantation (comparative alignment planning across flexion angles) No postoperative timepoints	Virtual implantation (patient-derived anatomy); postoperative rotational verification not applicable	Trochlear angle difference from native Trochlear under/overstuffing (relative to native) Trochlear sulcus ML position (lateralization/medialization across flexion)	All techniques understuffed the PF joint; MA showed greater deviations than KA (reported significant differences)	NR	KA restored native trochlear groove orientation better than MA in this morphometric model; clinical relevance remains uncertain
Wen et al^28^	Skyline (axial) and lateral radiographs 6 months postoperatively	No (not assessed)	Patellar tilt angle (°) Patellar height (BPI)	Change of lateral PF angle: KA 1.7° (SD 4.9°) vs MA 4.2° (SD 4.9°)	p < 0.01	KA showed significantly smaller tilt change

BPI, Blackburne-Peel Index; CDI, Caton-Deschamps Index; ISI, Insall-Salvati Index; NR, not reported; NS, not significant.

### Domain A: PF symptoms and PF-specific PROMs

Only one head-to-head comparative study reported PF-specific PROMs/symptoms (Table II). Parente et al^26^ compared inverse restricted KA with modified MA and assessed anterior knee pain and PF-specific outcomes including the Kujala score; no significant differences were observed between alignment approaches for these PF symptom-related endpoints. However, PF-specific PROM evidence was limited to a single study and is insufficient to support definitive patient-centred conclusions.

### Domain B: PF-related interventions and complications

Across six studies reporting PF-related interventions and complications, no consistent signal of higher PF-related intervention or adverse event burden with KA was identified compared with MA (Table II).

Four head-to-head studies reported LRR as a PF-related intervention. In a RCT, Sun et al^27^ directly evaluated LRR and reported a lower LRR rate in the KA group than in the MA group at 12 months. In a comparative cohort, King et al^30^ included LRR among PF-related interventions and did not report an excess LRR burden in the KA group. A large retrospective Chinese comparative study (Wen et al)^28^ also reported LRR frequency alongside range of motion and the Oxford Knee Score (OKS)^38,39^ during early follow-up, providing additional comparative evidence on PF-related interventions. Finally, in a bilateral, computer-assisted RCT, McEwen et al^29^ reported fewer soft-tissue releases overall in KA than in MA, while LRR itself was infrequent. Taken together, the available head-to-head evidence does not suggest that KA increases the need for LRR compared with MA.

Short-term safety signals were addressed in comparative cohort studies. King et al^30^ reported no meaningful between-group differences in early postoperative complications or revision events, including PF-related causes. Similarly, RCTs reporting adverse events did not identify an increased PF complication signal with KA. McEwen et al^29^ documented postoperative complications and secondary procedures in a bilateral RCT and did not report a higher complication burden with KA; Sun et al^27^ likewise reported comparable short-term complications between groups while observing a lower LRR rate with KA. Longer-term safety was evaluated in RCT follow-ups: Dossett et al^31^ reported no between-group differences in major or minor reoperations at a mean 13-year follow-up, and a ten-year randomized trial reported no differences in long-term clinical or radiological outcomes between KA and MA, with secondary patellar resurfacing being infrequent.^32^ Overall, across short- and long-term head-to-head studies, no consistent signal of increased PF-related complications or revision with KA was observed, although individual studies were not designed or powered to detect modest differences in uncommon PF events.

### Domain C: PF imaging / radiological measures

Ten studies reported PF imaging or radiological outcomes (Table III). Overall, imaging findings were mixed, and when differences were detected, they were not consistently linked to worse clinical outcomes. These imaging/radiological measures should be interpreted as surrogate outcomes, as their correlation with PF symptoms and PF-related interventions is uncertain and was inconsistently examined across studies. Several studies suggested small differences in early PF tracking surrogates after KA. For example, Nagai et al^35^ reported greater patellar shift at 30° flexion in KA than in MA at 12 months; importantly, patients with this shift were asymptomatic and patient-reported outcomes were comparable. Koh et al^33^ found a higher incidence of lateral patellar tilt early after surgery in KA, but this difference diminished by two years, with similar PROMs (OKS, Knee Society Score, 36-Item Short-Form Health Survey questionnaire)^40^ and satisfaction between groups.

Other imaging-focused comparative studies suggested that KA may preserve or restore PF-related measures towards a patient-specific baseline. Kim et al^34^ reported that the patellar tilt angle after KA remained closer to the preoperative status than after MA, while clinical scores were similar between groups. Elkadi et al^36^ evaluated multiple patellar height indices (Insall–Salvati Index,^41^ Caton–Deschamps Index,^42^ Blackburne–Peel Index)^43^ and reported better maintenance/improvement of patellar height measures with KA, noting that the clinical relevance of these radiological differences remains uncertain. Wen et al^28^ compared several PF imaging parameters (including the Blackburne–Peel Index and PF angles) and also reported clinical outcomes such as range of motion and the OKS, contributing additional early comparative data. Orsi et al^37^ used patient-derived trochlear morphology with virtual implantation to compare KA and MA, showing that KA deviated less from the native trochlear orientation than MA, while underscoring that the clinical relevance of these morphometric surrogates remains uncertain without corresponding symptom or intervention differences. Importantly, reporting of axial-plane alignment or postoperative rotational verification was inconsistent and uncommon, and no included study provided systematic CT-based postoperative component rotational assessment. This substantially limits inference regarding rotation-sensitive PF outcomes.

### Summary of mapped evidence

When restricted to direct KA-versus-MA comparative studies, the available literature was characterized by a marked imbalance across outcome domains: PF-specific patient-centred evidence was sparse (Domain A: one study), whereas imaging outcomes were more commonly assessed (Domain C). Across Domains A and B, no consistent harm signal was identified, but the available evidence remains insufficient to determine the definitive PF-specific clinical impact of KA versus MA.

## Discussion

The most important finding of this review is the limited patient-centred PF evidence in head-to-head KA–MA studies, with PF-specific PROMs reported in only one study.^26^ Although no consistent signal of worse clinically meaningful PF outcomes with KA was identified, PF symptoms and PF-specific PROMs were reported in only a very limited number of studies, precluding definitive patient-centred conclusions. PF-related interventions and complications – arguably the most clinically intuitive indicators of PF problems – were generally not reported more frequently with KA, and some studies reported numerically fewer PF-related procedures (e.g. LRR) with KA. Better restoration of patient-specific femoral axes with KA could plausibly improve patellar tracking and reduce lateral retinacular tension, thereby reducing the need for LRR. However, this mechanistic link remains unproven because postoperative rotational verification and mediation analyses were not performed, and LRR is highly dependent on technique and surgeon-specific indications. PF imaging outcomes were most commonly reported, but differences in surrogate radiological measures were not consistently accompanied by worse symptoms or higher PF-related intervention rates, highlighting a dissociation between imaging surrogates and patient-relevant outcomes. Importantly, patellar tracking after TKA is highly sensitive to axial-plane factors, particularly femoral component rotation (often considered in relation to the transepicondylar axis (TEA)–posterior condylar axis (PCA) relationship) and tibial component rotation; across included head-to-head studies, postoperative rotational alignment was inconsistently assessed and systematic CT-based verification was not reported, representing a major confounder when interpreting PF outcomes. Accordingly, the absence of a consistent harm signal should not be interpreted as excluding PF risk in rotation-sensitive situations. Interpretation is further limited by heterogeneity in KA variants (unrestricted, restricted/adjusted, and inverse-restricted), implant/trochlear design, and patellar resurfacing strategy, all of which are plausible effect modifiers for PF outcomes.

Prior systematic reviews that synthesized broad and heterogeneous evidence (including clinical, imaging, simulation, and cadaveric studies) have generally not identified a clear increase in PF problems with KA.^18^ However, for clinicians, studies that directly compare KA and MA under comparable clinical conditions may provide more directly interpretable evidence than broader syntheses that combine heterogeneous study designs. In this context, our focused scoping review adds value by isolating and mapping the available direct-comparison evidence on PF outcomes after KA versus MA, thereby clarifying what is supported by head-to-head data – and what remains insufficiently studied. In keeping with this broader literature, Courtney et al^19^ reported no difference in overall complication rates between KA and conventional TKA in randomized comparisons (3.9% vs 4.4%); although PF problems were the most common single reason for revision, the absolute frequency was low (1.2% of KA TKAs). Similarly, a network meta-analysis by Miura et al^20^ comparing KA, restricted KA, and MA reported little to no difference in revision risk between MA and KA, with comparable range of motion and a small PROM advantage for KA. Together with the present PF-focused head-to-head mapping, these findings indicate that no consistent harm signal has yet been identified, although the current evidence remains insufficient to determine the definitive PF-specific clinical impact of KA relative to MA.

Two plausible mechanisms may help explain why KA did not show a consistent signal of worse PF outcomes in head-to-head comparisons. First, KA may reduce lateral anterior femoral ‘overstuffing/overhang’, potentially lowering lateral retinacular tension and facilitating patellar tracking.^44-46^ Second, KA may promote a more physiological tibiofemoral kinematic pattern, including medial pivot motion, which could secondarily support patellar tracking.^47,48^ These hypotheses warrant direct testing, as causal links between these mechanistic factors and PF symptoms have not been consistently established in comparative clinical studies.

From a practical standpoint, these results suggest that isolated differences in PF radiological surrogates (e.g. tilt/shift, patellar height indices, and trochlea-related measures) should not be overinterpreted as definitive evidence of PF dysfunction in the absence of concordant symptoms or increased PF-related interventions. Accordingly, radiological findings are best interpreted alongside patient-centred outcomes (PF symptoms/PROMs) and PF-related interventions, rather than as stand-alone indicators of PF dysfunction. At the same time, the evidence base remains limited where it matters most to patients and clinicians: PF-specific symptom measures and PF-specific PROMs were rarely prespecified or systematically reported, and potential effect modifiers, such as patient phenotype (e.g. valgus alignment or advanced PF degeneration), implant design, component rotational strategy, and patellar resurfacing, were variably addressed. Future comparative trials and high-quality observational studies should therefore incorporate standardized PF symptom outcomes (e.g. anterior knee pain scales and PF-specific PROMs such as the Kujala score or equivalent) alongside global knee scores, and report PF-related interventions/complications using consistent definitions (e.g. indication and timing for LRR, criteria for PF instability/maltracking, patellar clunk/crepitus, and secondary PF procedures). Longer follow-up is also essential because PF complaints may emerge beyond the early postoperative period and PF-related reoperations are inherently time-dependent outcomes.

Outcomes specific to the PF joint (PFJ) may not be routinely reported in alignment studies for several practical and methodological reasons. Trials powered to detect a minimal clinically important difference in PFJ-related pain scores or PFJ-specific PROMs would typically require large sample sizes, because PFJ symptoms and PFJ-related interventions occur relatively infrequently and are influenced by multiple co-factors. In addition, widely used generic knee PROMs (e.g. the OKS and Forgotten Joint Score)^49^ capture overall function and satisfaction reasonably well, and investigators may therefore consider PFJ-specific PROMs less essential for primary endpoint selection. Standardized PFJ outcome reporting is further complicated by heterogeneity in how KA is executed (unrestricted, restricted/adjusted, and inverse-restricted variants with different boundaries), differences in implant and trochlear geometry, variation in technology and technique (manual, navigation, robotics, and soft-tissue balancing philosophy), and the frequent absence of postoperative verification – particularly for axial-plane rotational alignment. Collectively, these factors limit comparability across studies and likely contribute to the persistent under-reporting of PFJ-specific symptoms and PROMs.

This study has limitations inherent to scoping reviews and to the available evidence. First, we performed a descriptive synthesis without meta-analysis because of heterogeneity in KA/MA implementation, PF outcome definitions, and imaging protocols; therefore, the review maps patterns rather than providing pooled effect estimates. Second, restricting inclusion to English-language clinical comparative studies may have excluded relevant evidence. Third, PF outcomes were variably reported and rarely prespecified – particularly PF-specific PROMs – limiting inference and precluding robust conclusions about symptom-related endpoints. Imaging-based comparative studies without clinical follow-up primarily inform surrogate outcomes and therefore have limited direct clinical interpretability. In addition, the complete absence of systematic postoperative component rotational verification and the substantial heterogeneity in patellar resurfacing strategy, implant design, and KA implementation (e.g. unrestricted, restricted, and inverse restricted variants) further limit interpretation of PF-specific effects. In addition, substantial heterogeneity across studies limits interpretation: KA implementation, implant/trochlear design, and patellar resurfacing strategy varied widely and were incompletely reported in some cohorts. These factors are plausible effect modifiers for PF outcomes, and the small number of studies per stratum precluded subgroup analyses; therefore, findings should be interpreted as an evidence map rather than comparative estimates within specific KA variants or surgical/implant strategies. Finally, although we report overall study-level risk-of-bias judgements to support interpretation, findings should still be viewed as an evidence map rather than a graded certainty of evidence, given heterogeneity and limited PF-specific patient-centred outcomes.

Current head-to-head comparative evidence is insufficient to determine the definitive PF-specific clinical impact of KA versus MA in primary TKA. Although no consistent harm signal has been identified, the evidence remains insufficient for firm patient-centred conclusions and should be considered hypothesis-generating. The principal contribution of this review is therefore to define the key evidence gaps in the current literature. Future studies should incorporate prespecified PF-specific PROMs, consistent definitions of PF-related interventions and complications, systematic evaluation of axial-plane alignment, and longer-term follow-up to clarify the true patient-centred PF impact of alignment strategy.

**Take home message**

- This focused scoping review found no consistent signal of increased patellofemoral harm with kinematic alignment compared with mechanical alignment in primary total knee arthroplasty, although the available evidence remains limited and heterogeneous.

- Clinically, the findings suggest that radiological differences in patellar tracking should not be interpreted in isolation from patient-reported symptoms and patellofemoral interventions.

- The review also identifies key priorities for future studies, particularly patellofemoral-specific patient-reported outcome measures, systematic assessment of component rotation, consistent complication reporting, and longer follow-up.

## Data Availability

The data that support the findings for this study are available to other researchers from the corresponding author upon reasonable request.
